# Extensive gene rearrangements in the mitochondrial genomes of two egg parasitoids, *Trichogramma japonicum* and *Trichogramma ostriniae* (Hymenoptera: Chalcidoidea: Trichogrammatidae)

**DOI:** 10.1038/s41598-018-25338-3

**Published:** 2018-05-04

**Authors:** Long Chen, Peng-Yan Chen, Xiao-Feng Xue, Hai-Qing Hua, Yuan-Xi Li, Fan Zhang, Shu-Jun Wei

**Affiliations:** 1 Department of Entomology, Nanjing Agricultural University, Nanjing, 210095 China; 2 Institute of Plant and Environmental Protection, Beijing Academy of Agriculture and Forestry Sciences, Beijing, 100097 China; 3 Department of Entomology, South China Agricultural University, Guangzhou, 510640 China

## Abstract

Animal mitochondrial genomes usually exhibit conserved gene arrangement across major lineages, while those in the Hymenoptera are known to possess frequent rearrangements, as are those of several other orders of insects. Here, we sequenced two complete mitochondrial genomes of *Trichogramma japonicum* and *Trichogramma ostriniae* (Hymenoptera: Chalcidoidea: Trichogrammatidae). In total, 37 mitochondrial genes were identified in both species. The same gene arrangement pattern was found in the two species, with extensive gene rearrangement compared with the ancestral insect mitochondrial genome. Most tRNA genes and all protein-coding genes were encoded on the minority strand. In total, 15 tRNA genes and seven protein-coding genes were rearranged. The rearrangements of *cox1* and *nad2* as well as most tRNA genes were novel. Phylogenetic analysis based on nucleotide sequences of protein-coding genes and on gene arrangement patterns produced identical topologies that support the relationship of (Agaonidae + Pteromalidae) + Trichogrammatidae in Chalcidoidea. CREx analysis revealed eight rearrangement operations occurred from presumed ancestral gene order of Chalcidoidea to form the derived gene order of *Trichogramma*. Our study shows that gene rearrangement information in Chalcidoidea can potentially contribute to the phylogeny of Chalcidoidea when more mitochondrial genome sequences are available.

## Introduction

A typical animal mitochondrial genome is composed of 13 protein-coding, 22 tRNA and two rRNA genes, and a major non-coding sequence called the control region^1^. The sequences and structural features of mitochondrial genomes, such as the secondary structure of RNA genes, gene content and gene arrangement, reflect differences in function and evolutionary pattern in different taxa^2,3^. As an increasing number of mitochondrial genomes have been obtained, comparative feature analysis has become feasible among and within certain groups^3^. Gene rearrangement is one of the most frequently investigated features in animal mitochondrial genomes^3–7^. Comparative studies have shown that gene arrangements are usually conserved across major lineages but may be rearranged within some groups^2,4,8^. In insects, gene rearrangement has been reported in most orders. Accelerated rates of gene rearrangement have been found particularly in species of hemipteroids (thrips, book lice, lice)^7,9–11^, Protura^12^ and Hymenoptera (wasps, ants and bees)^6,13–16^. It has been known that the gene order of mitochondrial genome contains phylogenetic signals since the seminal work of Sankoff, *et al*.^17^ and Boore, *et al*.^18^. However, no gene rearrangements are shared between insect orders^3^. Examining gene rearrangement within lower taxonomic lineages of insects is expected to shed light on the evolution of these groups^5,19^.

Comparative studies may also contribute to understanding the forces that drive gene rearrangement. Gene rearrangements have been hypothesized to be correlated with parasitic life histories in the Hymenoptera^20,21^ and to some characteristics, such as body size and developmental time^22^. Frequent gene rearrangements have been observed in apocritan Hymenoptera based on broad examinations of whole or partial mitochondrial genome sequences^13,23^. Moreover, it has been reported that gene rearrangement was accelerated in the mitochondrial genomes of Apocrita^7,24,25^. However, no tight association was found between an increased rate of mitochondrial gene arrangement and the evolution of parasitism in an analysis of the characterization of 67 mitochondrial tRNA gene rearrangements in the Hymenoptera^16^.

**Figure 1:**
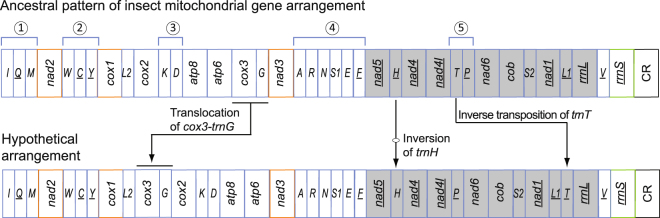
Ancestral arrangement of insect mitochondrial genes and types of gene rearrangement. The numbers 1 to 5 in circular indicate the five tRNA clusters. Transposition, inversion, inverse transposition were illustrated by comparing the ancestral pattern of insect mitochondrial gene arrangement and a hypothetical pattern.

Gene rearrangement patterns in the Hymenoptera are usually complicated and variable compared with those in most other insect orders^24,25^. Rearrangement of mitochondrial gene can be described by transposition, inversion, inverse transposition and TDRL (tandem duplication random loss) (Fig. 1)^3,26^. Bernt *et al*.^27^ introduced a movement of TDRL to describe the duplication of multiple contiguous genes and the successive random loss of one of the two copies^27^. In the Hymenoptera, rearrangements of tRNA genes usually occurred around the five gene clusters (Fig. 1). Inversion of *trnH* from the minority strand (heavy strand in mammal mitochondrial genomes) to the majority strand (light strand in mammal mitochondrial genomes, on which more genes are encoded) has also occurred multiple times in the Braconidae (Hymenoptera)^26^. The rate of rearrangement of protein-coding genes is lower than that of tRNAs in the Hymenoptera. Rearrangement of protein-coding gene has been reported in limited species of Agaonidae (Chalcidoidea)^14^, Aulacidae (Evanioidea)^28^, Trigonalyidae (Trigonalyoidea)^29^, Pteromalidae (Chalcidoidea)^30^, Ichneumonidae (Ichneumonoidea)^16^, Braconidae (Ichneumonoidea)^15^ and Bethylidae (Chrysidoidea)^13^.

In the Chalcidoidea (Hymenoptera: Apocrita), unusually high rates of gene rearrangement, including not only tRNA genes but also protein-coding genes, have been found^14,30^. Combined with the diverse lifestyles among species of this group, this high rearrangement rate provides suitable materials for examining the evolution of gene rearrangement. Presently, only a few complete or partial mitochondrial genomes are known from Chalcidoidea, including those from *Megaphragma*^31^, *Nasonia*^30^, and *Philotrypesis*^14^. The Trichogrammatidae (Chalcidoidea) are small egg parasitoids with a short developmental duration and are one of the most important groups used in the biological control of insect pests. The parasitoids in this family can parasitize the eggs of about 10 orders. However, no complete mitochondrial genome has previously been sequenced from members of this family except for the mitochondrial genome sequence from *Megaphragma*^31^. Increasing knowledge of the mitochondrial genomes of egg parasitoids will provide further insight into their higher-level phylogeny and the evolution of their life histories.

In the study, we sequenced two mitochondrial genomes from *Trichogramma ostriniae* and *Trichogramma japonicum*. We found novel and extensive gene rearrangements in both species compared with the ancestral insect mitochondrial genome. Phylogenetic relationships within the Chalcidoidea were reconstructed using mitochondrial genome sequences and gene arrangement patterns.

## Results and Discussion

### Genome structure

The complete mitochondrial genome of *T*. *japonicum* (GenBank accession number: KU577436) and *T*. *ostriniae* (GenBank accession number: KU577437) were determined, with lengths of 15,962 bp and 16,472 bp, respectively. The sizes were well within the range found in other completely sequenced hymenopteran insects (from 15,137 bp in *Idris* sp. to 19,339 bp in *Cephus cinctus*)^32,33^ (Table S1). All typical animal mitochondrial genes and control regions were identified in both circular genomes (Table 1).

**Table 1:** Annotation of the *Trichogramma japonicum* and *Trichogramma ostriniae* mitochondrial genomes.

Gene	Strand	*Trichogramma japonicum*					*Trichogramma ostriniae*				
		Position	Size	INT	Start/stop codon		Position	Size	INE	Start/stop codon	
*trnW*	−	1–66	66	0			1–67	67	0		
*nad2*	−	67–1078	1014	0	ATA	T	68–1080	1014	0	ATA	TA
*trnQ*	−	1079–1146	68	17			1081–1148	68	63		
*trnY*	−	1164–1230	67	41			1212–1277	66	62		
*cox1*	−	1272–2807	1536	15	ATG	TAA	1340–2875	1536	1	ATG	TAA
*trnE*	+	2823–2889	67	25			2877–2942	66	2		
*trnF*	−	2915–2978	64	6			2945–3009	65	171		
*trnI*	−	2985–3051	67	3			3181–3247	67	0		
*trnS1*	−	3055–3113	59	67			3248–3307	60	151		
*trnN*	−	3181–3246	66	20			3459–3524	66	0		
*trnC*	−	3267–3335	69	52			3525–3592	68	106		
*cox3*	−	3388–4179	792	35	ATG	TAA	3699–4490	792	24	ATG	TAA
*atp6*	−	4215–4889	675	−7	ATG	TAA	4515–5189	675	−7	ATG	TAA
*atp8*	−	4883–5050	168	81	ATT	TAA	5183–5350	168	72	ATT	TAA
*trnD*	−	5132–5197	66	12			5423–5488	66	7		
*trnK*	+	5210–5279	70	14			5496–5565	70	9		
*cox2*	−	5294–5974	681	0	ATT	TAA	5575–6255	681	0	ATT	TAA
*trnL2*	−	5975–6040	66	31			6256–6321	66	29		
*nad5*	−	6072–7757	1686	1	ATA	TAA	6351–8033	1683	6	ATT	TAA
*trnH*	−	7759–7825	67	21			8040–8102	63	30		
*nad4*	−	7847–9190	1344	−7	ATG	TAA	8133–9476	1344	−7	ATG	TAA
*nad4l*	−	9184–9471	288	10	ATT	TAA	9470–9757	288	0	ATT	TAG
*trnT*	+	9482–9546	65	−1			9758–9821	64	−1		
*trnP*	−	9546–9611	66	6			9821–9885	65	13		
*nad6*	+	9618–10196	579	33	ATT	TAA	9899–10471	573	2	ATG	TAA
*cob*	+	10230–11369	1140	25	ATG	TAA	10474–11613	1140	19	ATG	TAA
*trnS2*	+	11395–11458	64	−2			11633–11696	64	−2		
*nad1*	−	11457–12392	936	0	ATT	TAA	11695–12630	936	0	ATT	TAA
*trnL1*	−	12393–12457	65	0			12631–12700	70	0		
*rrnL*	−	12458–13857	1400	0			12701–14067	1367	0		
*trnA*	−	13858–13922	65	14			14068–14131	64	10		
*trnG*	−	13937–14001	65	0			14142–14208	67	0		
*rrnS*	−	14002–14791	790	0			14209–14983	775	0		
*trnV*	−	14792–14857	66	−2			14984–15051	68	−1		
*trnR*	−	14856–14920	65	18			15051–15113	63	103		
*nad3*	−	14939–15301	363	0	ATA	TAA	15217–15576	360	0	ATA	TAA
*trnM*	−	15302–15369	68	0			15577–15642	66	0		
control region		15370–15962	593				15643–16472	830			

^+^ indicates the gene is coded on majority strand while ^−^indicates the gene is coded on minority strand. INT indicates the intergenic nucleotides. Positive values indicate intergenic nucleotides and negative values indicate overlapping nucleotides between two adjacent genes.

In the mitochondrial genome of *T*. *japonicum*, a total of 547 bp of intergenic nucleotides ranging from 1 to 81 bp were found in 17 locations. The longest intergenic spacer (81 bp) was found between *atp8* and *trnD*. In the mitochondrial genome of *T*. *ostriniae*, there were 870 bp intergenic spacer sequence distributed among 19 locations with lengths from 1 to 171 bp. The longest intergenic spacer (171 bp) was located between *trnF* and *trnI*. Long intergenic spaces have been identified in other insect mitochondrial genomes^13,34^ and were considered as possible results of gene rearrangement^29^.

Overlapping genes are very common in arthropod mitochondrial genomes^34–36^. In the mitochondrial genome of *T*. *japonicum*, a total of 19 bp of overlapping nucleotides were detected with a length from 1 to 7 bp, while in that of *T*. *ostriniae* there were 18 bp shared nucleotides in total, also ranging from 1 to 7 bp. In both species, the overlapping regions were found in the same five locations, i.e., *atp6*-*atp8*, *nad4*-*nad4l*, *trnT*-*trnP*, *trnS2*-*nad1* and *trnV*-*trnR*. The other 10 pairs of genes in *T*. *japonicum* and 13 pairs of genes in *T*. *ostriniae* were directly adjacent, without overlapping or intergenic nucleotides.

The sequences of both mitochondrial genomes are biased in nucleotide composition [(A + T)% > (G + C)%] (Table S2), which is common in mitochondrial genomes of suborder Apocrita (Hymenoptera)^19,37,38^. The parameters of AT skew and GC skew are frequently used to reveal the nucleotide-compositional behavior of mitochondrial genomes^39–41^. In both species, the AT skews of the majority strand were positive, while GC skews were negative, which indicated that the two mitochondrial genomes contained more A than T and more C than G nucleotides (Table S2), as reported for most hymenopteran insects^42^ (Table S1).

### Transfer RNA and ribosomal RNA genes

In total, 22 tRNA genes were interspersed throughout the *Trichogramma* mitochondrial genomes, of which four were coded on the majority strand while 18 were coded on the minority strand. The tRNA genes ranged from 59 bp (*trnS1* in *T*. *japonicum*) to 70 bp (*trnK* in *T*. *japonicum*), well within the range observed in other insects (Table 1). All tRNA sequences can be folded into the canonical cloverleaf secondary structure, except for *trnS1* which lacked the dihydrouridine (DHU) arm. A lack of the DHU arm in *trnS1* was found in the mitochondrial genomes of most insects^1,43^ and other metazoans^44^. Variations in the lengths of the variable loop, DHU and TΨC arms result in the different sizes observed in the tRNA sequences^45^. In total four mismatches (U-U in *trnY*, *trnW*, *trnG* and *trnC*) were found in *T*. *japonicum* and five (U-U in t*rnY*, *trnW*, *trnG*, *trnC* and *trnN*) in *T*. *ostriniae*. Mismatches were located mostly in the DHU and anticodon stems (Figure S1).

As with other insect mitochondrial genome sequences, the large and small ribosomal RNA genes (*rrnL* and *rrnS*) were encoded by the minority strand in the same location (between *trnL1*-*trnA* and *trnG*-*trnV*). In *T*. *japonicum*, the length of the *rrnS* gene was 790 bp with an A + T content of 87.72%, while the *rrnL* gene was 1400 bp with an A + T content of 88.36%. In *T*. *ostriniae* the length of the *rrnS* gene was 775 bp with an A + T content of 88.52%, while the *rrnL* gene was 1367 bp with an A + T content of 88.00%.

### Protein-coding genes and codon usage patterns

In both the *T*. *japonicum* and *T*. *ostriniae* mitochondrial genomes, 11 of 13 protein-coding genes were encoded by the minority strand, while two (*nad6* and *cob*) were encoded by the majority strand. All homologous protein-coding genes from the two species had the same length, except for *nad*3, *nad*6 and *nad5* (Table 1).

In the mitochondrial genome of *T*. *japonicum*, the total length of the protein-coding genes was 11,202 bp, accounting for 70.18% of the entire genome. The average A + T content of the 13 protein-coding genes was 83.08%, ranging from 76.04% (*cox1*) to 90.80% (*nad2*) for individual genes. In the mitochondrial genome of *T*. *ostriniae*, the total length of protein-coding genes was 11,190 bp, accounting for 67.93% of the entire genome. The average A + T content of the 13 protein-coding genes was 83.25%, ranging from 76.37% (*cox1*) to 91.30% (*nad2*) for individual genes (Table S2).

The predicted initiation codons are ATN, as in most other insect mitochondrial genomes^37,46^. In some cases, a given gene may have different start codons in different species. There were seven genes (*nad2*, *nad3*, *nad1*, *nad4L*, *nad5*, *cox2* and *atp8*) starting with ATG and five genes (*cox1*, *cob*, *nad4*, *atp6* and *cox3*) starting with ATA in both genomes. In *T*. *ostriniae*, *nad6* started with ATG, but in *T*. *japonicum* it started with ATA. All protein-coding genes terminated at the most common stop codon, TAA, in both genomes, except for *nad4l* in *T*. *ostriniae*, which stopped with TAG, and *nad2*, which stopped with T and TA in *T*. *ostriniae* and *T*. *japonicum*, respectively.

Codons with high A/T content were preferred in these two species, as in most insect mitochondrial genomes^47^. In both species of this study, Ala, Gly, Leu, Pro, Arg, Ser, Thr and Val were the most frequently used amino acids, and UUA (Leu) had the highest relative synonymous codon usage (RSCU) (Table S3). All remaining codons with RSCU > 2.00 preferred A/T in the third codon position.

### Control region

Complete control regions were found in both species. The length of the control region was 593 bp in *T*. *japonicum* and 830 bp in *T*. *ostriniae*, which was well within the range reported in insects^21,48^. The control region in both species was flanked by *trnW* and *trnM* with high A + T content (90.99% in *T*. *japonicum* and 89.03% in *T*. *ostriniae*).

The control region is believed to function in the initiation of replication and control of transcription of the mitochondrial genome^49^. This region is usually characterized by five conserved elements^8,50^ as reported in some insect mitochondrial genomes^15^. All of those elements could be identified in the mitochondrial genomes of *Trichogramma*, such as (1) a polyT stretch at the 5′ end of the control region; (2) a [TA(A)]n-like stretch following the polyT stretch; (3) a stem and loop structure (Figure S2); (4) a TATA motif and a G(A)nT motif flanking the stem and loop structure; and (5) a G + A-rich sequence downstream of the stem and loop structure. However, they were not in the typical orders and positions, as reported in some insect species^34^.

Concerted evolution is common in the insect control region, most obviously in species with repeat units in their control regions such as termites^51^ but also in species with non-tandemly repeated control regions such as thrips^52^. Repeat sequences were found in both species of *Trichogramma*, as have been reported in some other insects^15,40^. In *T*. *japonicum*, three 21-bp tandem repeats of “AGCCTCAAAAATCGGGGTTTT” and two 41-bp tandem repeats of “ATTATTATATAAATTATTTATATTTATATAAATATTTAATA” were found in the control region. In the three 21-bp tandem repeats, three mutations (“GCC” to “CTT”) in the first repeat region were present. The control region of *T*. *ostriniae* contained nine 21-bp tandem repeats with several mutations among repeat units (Figure S3). There was an 80-bp perfect repeat of TA in control region of *T*. *ostriniae*. The presence of repeat regions may inhibit DNA polymerase and could lead to the failure of sequencing in those regions^53,54^.

### Gene arrangement

**Figure 2:**
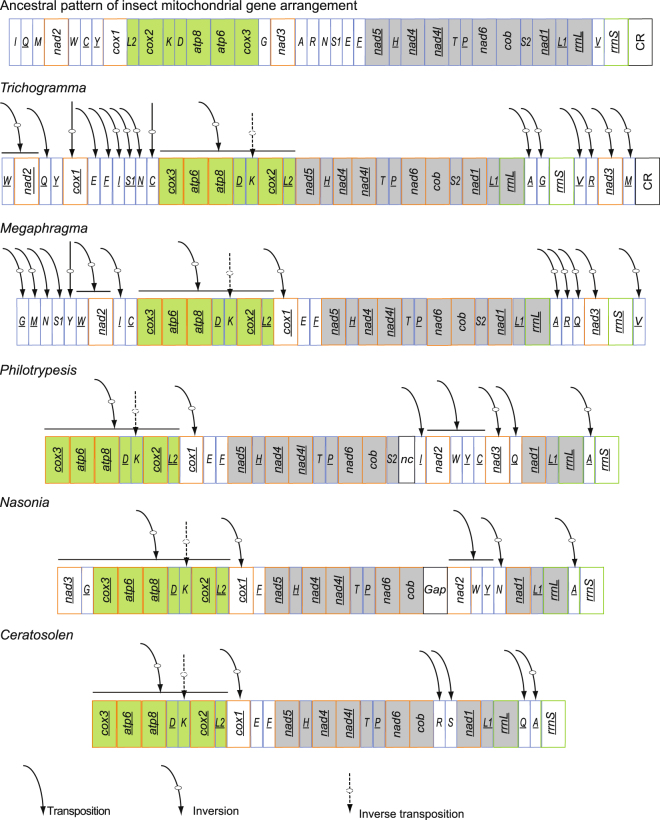
Mitochondrial genome organization and gene rearrangement in Chalcidoidea compared with the ancestral type of the insect mitochondrial genome. The gene order is linearized for easy view. The gene blocks with inversion are shown in green, while the conserved gene blocks are showed in grey. Genes nomenclature: *atp6* and *atp8*; ATP synthase subunits 6 and 8; *cob*: cytochrome b; *cox1*–*3*: cytochrome c oxidase subunits 1–3; *nad1*–*6* and *nad4L*: NADH dehydrogenase subunits 1–6 and 4 L; *rrnS* and *rrnL*: small and large subunit ribosomal RNA (rRNA) genes; Transfer RNA genes are denoted by a one-letter symbol according to the IPUC-IUB single-letter amino acid codes. L1, L2, S1 and S2 denote tRNAL (CUN), tRNAL (UUR), tRNAS (AGN) and tRNAS (UCN), respectively. CR indicate the control region.

In previously studied insect mitochondrial genomes, most rearranged genes were tRNA genes^16,25^. In the Hymenoptera, numerous rearrangements of protein-coding genes have been identified in several groups^13–15^. Compared with the putative ancestral pattern of the insect mitochondrial genome, dramatic gene rearrangements, not only in tRNA genes but also in protein-coding genes, were found in *Trichogramma* mitochondrial genomes. In total, 15 of 22 tRNA genes and seven of 13 protein-coding genes were rearranged in *Trichogramma* compared with the ancestral arrangement (Fig. 2). All genes in the mitochondrial genomes of the two *Trichogramma* species were encoded by the minority strand, rather than the majority strand, except for two protein-coding genes (*cob* and *nad6*) and four tRNA genes (*trnE*, *trnK*, *trnT* and *trnS2*).

Compared with the other sequenced mitochondrial genomes of Chalcidoidea, *cox1* was inverted separately in *Trichogramma*, not together with the gene block of *cox1*-*trnL2*. The protein-coding gene *nad2* did not change its relative position but changed direction compared with the ancestral type. The gene clusters between *cox2*-*atp8*, *nad3*-*nad5*, *nad2*-*cox1* and control region-*nad2* have been identified as the most frequently rearranged regions in mitochondrial genomes of Hymenoptera^13,19^, which also applied to *Trichogramma*. A novel tRNA gene cluster *trnE*-*trnF*-*trnI*-*trnS1*-*trnN*-*trnC* formed between *cox1* and *cox3*. The tRNA cluster *trnA*-*trnG* formed between two ribosomal RNA genes; this is also novel in the Hymenoptera, in which the *trnV* gene is typically located between them^30^; Although the conservation and inversion of large-scale gene blocks in *Trichogramma* was similar to other sequenced mitochondria genomes of Chalcidoidea, the rearrangement of protein-coding genes *nad2* and *cox1* as well as most tRNA genes are novel.

### Phylogenetic relationships among families of Chalcidoidea

Currently, mitochondrial genomes have been sequenced from three families of Chalcidoidea in seven species. Phylogenetic relationships among the seven species were reconstructed based on protein-coding genes of the mitochondrial genome.

**Figure 3:**
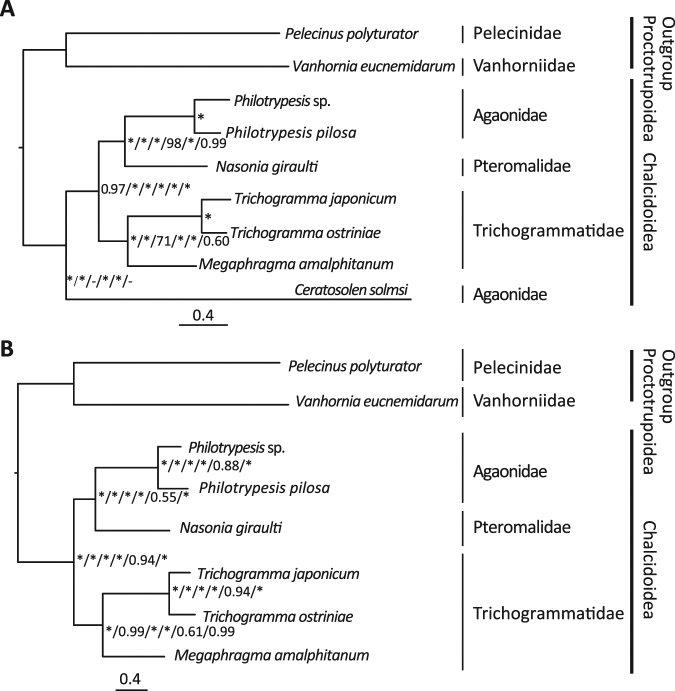
Phylogenetic relationships within Chalcidoidea based on the nucleotide and amino acids sequences of 13 protein-coding genes. (**A**) Seven species of Chalcidoidea with mitochondrial genome sequence were included. (**B**) *Ceratosolen solmsi* was excluded from analysis to avoid the potential long-branch attraction. The values separated by “/” near the nodes represent support rates of corresponding node. The six values indicate the posterior probabilities of Mrbayes analysis based on nucleotide and amino acids sequences, bootstrap probabilities of IQ-TREE analysis based on nucleotide and amino acids sequences, posterior probabilities of Phylobayes analysis based on nucleotide and amino acids sequences. “*” indicates the 1.0 Bayesian posterior probability and 100 bootstrap value and “−” indicates the absence of the node in corresponding analysis.

The results showed that the species *Ceratosolen solmsi* from Agaonidae was not clustered with two other species of this family, even when the CAT model was used to avoid among-site rate heterogeneities (Fig. 3A). This species had a long branch compared to other species of ingroup, as shown in the original study of the mitochondrial genome of this species^14^. We predict that the inferred polyphyly of Agaonidae might be caused by long-branch attraction in *C*. *solmsi*. The Hymenoptera has been shown to be a group with both rapidly and slowly evolving mitochondrial genomes^32^. Long branches have been identified in *Cephalonomia gallicola* (Chrysidoidea: Bethylidae), *Wallacidia oculata* (Vespoidea: Mutillidae)^13,55^ and *Primeuchroeus* spp. (Aculeata: Chrysididae)^55^. Identification of other species with long branches may help to improve phylogenetic inference of relationships within Hymenoptera.

By removing the outlier species *C*. *solmsi* from the analysis, a well-supported phylogenetic relationship among three families of Chalcidoidea was generated (Fig. 3B). The Agaonidae and Pteromalidae formed one lineage, which was then sister to Trichogrammatidae. This is congruent with the currently supported phylogeny of Chalcidoidea^56^.

We also used gene arrangement to reconstruct phylogenetic relationships among the three families. The inferred topology is identical to the one generated from gene sequences (Appendix S1). Our initial work indicates that gene rearrangements of the mitochondrial genome may provide valuable information for recovering phylogenetic relationships within Chalcidoidea. More representative mitochondrial genomes from different groups are needed to validate our assumption.

### Ancestral gene order in Chalcidoidea

**Figure 4:**
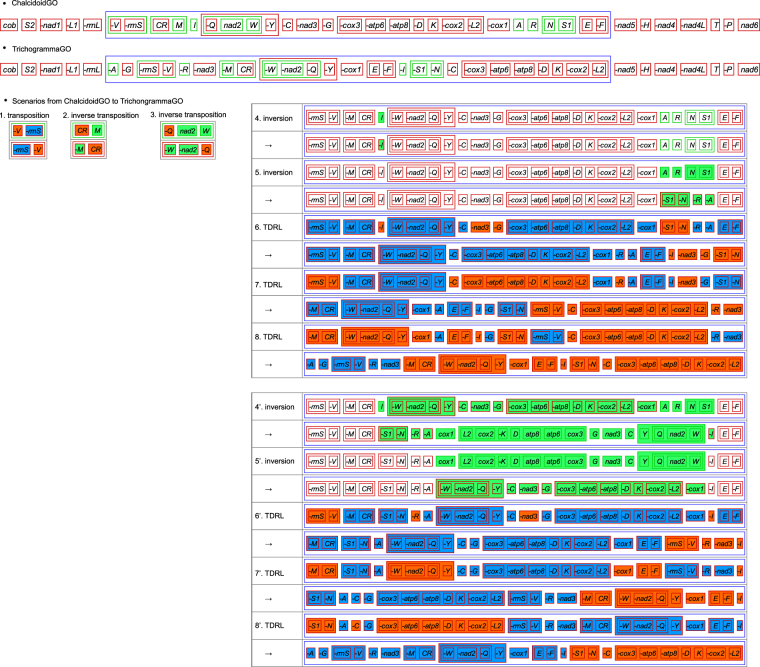
Evolution of gene order in mitochondrial genomes *Trichogramma* explained by software CREx. In total eight rearrangement operations occurred from presumed ancestral gene order of Chalcidoidea (ChalcidoidGO) to form the derived gene order of *Trichogramma* (TrichogrammaGO). Two alternative sets of scenarios were found, i.e. operations 4–8 and operations 4′–8′.

Large scale gene rearrangements were also found in other sequenced mitochondrial genomes of Chalcidoidea^14,30^ (Fig. 2). Babbucci *et al*.^23^ showed a gene order (GO) named ant1GO as the plesiomorphic GO for Hymenoptera^23^. Simultaneous rearrangement of large groups of genes has been considered as the initial step of gene rearrangement in the extremely rearranged mitochondrial genomes of *Cotesia vestalis*^15^. Rearrangement of large groups of genes was common in species of Chalcidoidea (Fig. 2). By comparisons, a conserved segment of “*trnE* -*trnF* -*nad5* -*trnH* -*nad4* -*nad4L trnT* -*trnP nad6 cob*” was found among ancestral pattern of insect mitochondrial gene arrangement (PanGO), ant1GO and chalcidoid species of *Megraphragma*^31^, *Philotrypesis*^14^ and *Ceratosolen* (Fig. 2), and a segment of “*trnS2* -*nad1* -*trnL1* -*rrnL*” was shared by ant1GO, PanGo and *Trichogramma* and *Megaphragma*^31^ (Fig. 2). Based on the inferred phylogenetic relationships that Trichogrammatidae (*Trichogramma* + *Megaphragma*) is sister to (Agaonidae + Pteromalidae) (Fig. 3B), a segment of “*trnE* -*trnF* -*nad5* -*trnH* -*nad4* -*nad4L trnT* -*trnP nad6 cob trnS2* -*nad1* -*trnL1* -*rrnL*” is more plausible as the ancestral pattern of Chalcidoidea. Within Chalcidoidea, an inverted segment “-*cox3* -*atp6* -*atp8* -*trnD trnK* -*cox2* -*trnL2* -*cox1*” compared with PanGO was shared by all analyzed taxa except for *Trichogramma*. A bigger inverted continuous segment with -*nad3* and -*trnG* was found in *Nasonia* species^30^ (Fig. 2), which strongly support the ancestral pattern of “-*nad3* -*trnG* -*cox3* -*atp6* -*atp8* -*trnD trnK* -*cox2* -*trnL2* -*cox1*” for Chalcidoidea. For the tRNA clusters, the pattern of “-D k” was conserved across all analyzed species of Chalcidoidea (Fig. 2). Since there is no conserved pattern in tRNA clusters “*trnI* -*trnQ trnM*”, “*trnW* -*trnC* -*trnY*” and “*trnA trnR trnN trnS trnE* -*trnF*”, those in ant1GO were presumed as the ancestral pattern of gene order in Chalcidoidea (ChalcidoidGO in Fig. 4).

### Rearrangement pathway of Trichogramma

We inferred the evolution of gene arrangement in *Trichogramma* using CREx by comparing the common intervals between ChalcidoidGO and *Trichogramma* gene order (TrichogrammaGO) (Fig. 4). Four operations were considered in CREx, i.e., transpositions, inversions, inverse transpositions, and TDRL. The CREx identified eight operations in the evolution of gene arrangement in *Trichogramma*, including one transposition (operation 1 in Fig. 4, referring to *rrnS*), two inverse transpositions (operations 2 and 3 in Fig. 4, referring M and W, respectively), two inversions (operations 4–5 in Fig. 4) and three TDRLs (operations 6–8 in Fig. 4).

There are two sets of alternative scenarios in operations 4–8. The first set of scenarios refers to inversions of *trnI* and tRNA cluster from *trnA* to *trnF*, followed by three TDRLs (operations 4–8 in Fig. 4), while the other set refers to inversion of two large gene segments including both tRNA and protein-coding genes, followed by three TDRLs (operations 4′–8′ in Fig. 4). The presence of intergenic spacers located in the position involved in rearrangements and presence of remnant of the genes that changed positions within intergenetic spacers will provide evidence to choose the plausible rearrangement pathways^23,57^. However, we did not find obvious evidence to support one set of scenarios. Mapping the gene rearrangement patterns on the inferred phylogenetic tree may help to validate the scenarios using MLGO^58^ or TreeRex^59^. However, the missing of genes in most species limited the usage of this approach. Rearrangement of tRNA genes are believed to be more frequent than that of large segment^3,16^. Thus, we presumed that scenarios 4–8 are more plausible than 4′–8′ in rearrangement of *Trichogramma* mitochondrial genomes. However, we could not exclude other pathways due to the computational complexity in gene order reconstruction and the algorithms implemented in CREx^27,57^.

## Methods

### DNA extraction

Specimens of *T*. *japonicum* and *T*. *ostriniae* were reared in the Insectary of Nanjing Agricultural University. DNA was extracted from individual wasps using a Wizard^®^ Genomic DNA Purification system (Promega) according to the manufacturer’s protocols and stored at −20 °C. The specimens are deposited in the Laboratory of Molecular Ecology and Evolution of Nanjing Agricultural University (*T*. *ostriniae*: NJAUHymTrich001; *T*. *japonicum*: NJAUHymTrich003).

### Mitochondrial genome amplification and annotation

Two short fragments (518 bp) of the *cox1* gene were amplified using primer set 1718-COI-F/2191-COI-R (Simon *et al*. 1994) for *T*. *japonicum* and *T*. *ostriniae*. PCR products were purified and sequenced directly using the Sanger method at Shanghai Majorbio Bio-pharm Co., Ltd. (Shanghai, China). According to the obtained sequences, specific PCR primers (Table S4) for each species (*T*. *japonicum*: Tj-COI-F/Tj-COI-R and *T*. *ostriniae*: T0-COI-F/To-COI-R) were designed to amplify the remaining genome by long PCR as a single fragment, using the manufacturer’s rapid PCR protocol. The reaction mixture was composed of 1 μL Tks Gflex DNA Polymerase (Takara), 25 μL buffer, 1 μL of each primer (20 μM), 3 μL of DNA with water added to bring a total volume of 50 μL. The cycling conditions were 94 °C for 1 min, 30 cycles of 98 °C for 10 s, 55 °C for 15 s and 68 °C for 10 min. The PCR products were sequenced on an Illumina HiSeq. 2500 platform at Shanghai Majorbio Bio-pharm Co., Ltd. (Shanghai, China). Sequencing libraries for the long PCR fragments were prepared using a TruSeq DNA Sample Prep Kit (Illumina) following the manufacturer’s instructions. Libraries were purified with Certified Low Range Ultra Agarose (Bio-Rad), quantified on a TBS380 fluorometer (Invitrogen), pooled and sequenced using a HiSeq SBS Kit V4 with tag sequences generating paired-end reads (read length: 250 bp).

Raw sequencing data were generated by Illumina base calling software CASAVAv1.8.2 (http://support.illumina.com/sequencing/sequencing_software/casava.ilmn), and sequences containing adaptors or primers were identified by SeqPrep (https://github.com/jstjohn/SeqPrep). Sickle (https://github.com/najoshi/sickle)^60^ was applied to conduct reads trimming with default parameters to obtain clean data for this study. In addition, SOAPdenovo (http://soap.genomics.org.cn/, v2.05)^61^ was used to perform genome assembly with multiple Kmer parameters and assess the assembly results. GapCloser software^61^ (downloaded from SOAPdenovo website) was subsequently applied to fill the remaining local inner gaps and correct single-base polymorphisms for the final assembly results.

The tRNA genes were initially identified using tRNA-scan SE 1.21 (http://lowelab.ucsc.edu/tRNAscan-SE/)^62^, with the following parameters: source = Mito/Chloromast, and genetic code = Invertebrate Mito. Twenty of the 22 typical animal mitochondrial tRNA genes were identified. The remaining two tRNA and two rRNA genes were confirmed by the MITOS WebServer using invertebrate mitochondrial genetic code (http://mitos.bioinf.uni-leipzig.de/index.py) (Bernt *et al*., 2013). ORFinder (http://www.ncbi.nlm.nih.gov/gorf/orfig.cgi) was used to identify protein-coding genes, specifying the invertebrate mitochondrial genetic code.

### Genome feature analysis

Base composition, codon usage, Relative Synonymous Codon Usage (RSCU) values and nucleotide substitution were analyzed using MEGA6^63^. GC and AT asymmetries were calculated according to the formulas used in a previous study^40^. AT- and GC-skews were measured for the majority strand using the formulas AT skew = (A − T)/(A + T) and GC skew = (G − C)/(G + C). The tandem repeats in the control region were predicted using the Tandem Repeats Finder available online (http://tandem.bu.edu/trf/trf.html)^64^.

### Phylogenetic analysis

Phylogenetic relationships among three families of Chalcidoidea with sequenced mitochondrial genomes were reconstructed. A phylogenetic tree was reconstructed based on the nucleotide sequences and amino acid sequences of the 13 protein-coding genes. Nucleotide sequences were aligned by codon using MAFFT version 7.205^65^. Phylogenetic relationships were reconstructed with MrBayes version 3.2.5^66^, IQ-TREE web server^67^ and PhyloBayes-MPI^68^. In MrBayes analyses, matrices were partitioned by codon position. Then, we used PartitionFinder version 1.1.1^69^ to determine the best partition and substitution models. Four independent Markov chains were run for 10 million metropolis-coupled generations, with tree sampling every 1000 generations and a burn-in of 25%. In IQ-TREE analyses, the number of bootstrap replicates was set to 1000 with CAT model (C20 + 4 G)^70^. The CAT-GTR model was applied in PhyloBayes analyses with independent Markov chain runs of 8000 and a burn-in of 1000 and subsample of 10 trees. *Pelecinus polyturator* (Proctotrupoidea: Pelecinidae)^55^ and *Vanhornia eucnemidarumI* (Proctotrupoidea: Vanhorniidae)^71^ were chosen as outgroups according to previously inferred phylogenetic relationships among major lineages of Apocrita^13,55^.

We also inferred phylogenetic relationships among the three families of Chalcidoidea based on gene arrangement patterns. Phylogenetic relationships were inferred using a Maximum Likelihood (ML) method based on gene-order data implemented in the MLGO web server^58^. We excluded *C*. *solmsi* from the analysis due to missing genes and the inclusion of other representatives from the family Agaonidae.

### Gene organization analysis

The evolutionary pathways of gene arrangement in *Trichogramma* were estimated by CREx (Common Interval Rearrangement Explorer)^27^. The heuristic method based on common interval was used to determine genome rearrangement scenarios between presumed ancestral gene order of Chalcidoidea and that of *Trichogramma*. Gene rearrangement pattern were mapped to the phylogenetic tree using MLGO web server^58^. The ChalcidoidGO was used as outgroup.

### Data availability statement

The data were deposited into GenBank under accession numbers: KU577436 and KU577437.

## Conclusion

The two mitochondrial genomes sequenced in our study from *Trichogramma* add to our knowledge of the mitochondrial genomes of Hymenoptera. Compared with those of other related insects, the mitochondrial genomes of *Trichogramma* were significantly rearranged, not only in tRNA genes but also in many protein-coding genes. Congruent tree topologies were recovered using gene sequences, including nucleotides and amino acids. The specific gene order in mitochondrial genomes of these species indicated that gene rearrangement information may represent potentially valuable data for phylogenetic analyses of Chalcidoidea. The availability of the complete mitochondrial genomes of *Trichogramma* provides information for development of genetic markers to study community ecology, population biology and evolution in this species complex.

## Electronic supplementary material

Supplemental information
