# Supplementary material for: Extensive gene rearrangements in the mitochondrial genomes of two egg parasitoids, Trichogramma japonicum and Trichogramma ostriniae (Hymenoptera: Chalcidoidea: Trichogrammatidae)
Source: Sci Rep. 2018 May 4;8:7034. doi: 10.1038/s41598-018-25338-3 (PMC5935716; doi:10.1038/s41598-018-25338-3)

Extensive gene rearrangements in the mitochondrial genomes of two egg parasitoids *Trichogramma* *japonicum* and *Trichogramma* *ostriniae* (Hymenoptera: Chalcidoidea: Trichogrammatidae)

Long Chen^1^, Peng-Yan Chen^2,3^, Xiao-Feng Xue^1^, Hai-Qing Hua^1^, Yuan-Xi Li^1^, Fan Zhang^2^, Shu-Jun Wei^2^

^1^ Department of Entomology, Nanjing Agricultural University, Nanjing 210095, China;

^2^ Institute of Plant and Environmental Protection, Beijing Academy of Agriculture and Forestry Sciences, Beijing 100097, China

^3^ Department of Entomology, South China Agricultural University, Guangzhou 510640, China

**Table S1 General features of the mitochondrial** genomes sequenced in Hymenopteran

| Species | Superfamily | Accession number | Length(bp) | A+T content (%) | AT-skew | GC-skew |
| --- | --- | --- | --- | --- | --- | --- |
| *Apis cerana* | Apoidea | NC_014295 | 15895 | 84.0 | 0.008 | -0.217 |
| *Apis florea* | Apoidea | NC_021401 | 17694 | 86.1 | 0.038 | -0.270 |
| *Apis mellifera* | Apoidea | KM458618 | 16336 | 84.6 | 0.021 | -0.267 |
| *Bombus hypocrita* | Apoidea | NC_011923 | 15468 | 85.3 | 0.003 | -0.320 |
| *Bombus ignitus* | Apoidea | NC_010967 | 16434 | 86.8 | 0.003 | -0.272 |
| *Colletes gigas* | Apoidea | NC_026218 | 15881 | 86.2 | -0.024 | -0.076 |
| *Hylaeus dilatatus* | Apoidea | NC_026468 | 15472 | 85.8 | 0.032 | -0.239 |
| *Melipona bicolor* | Apoidea | NC_004529 | 14422 | 86.7 | 0.016 | -0.251 |
| *Melipona scutellaris* | Apoidea | NC_026198 | 14862 | 86.8 | 0.012 | -0.249 |
| *Ceraphron* sp*.* | Ceraphronoidea | KJ570858 | 14947 | 80.0 | 0.082 | -0.394 |
| *Conostigmus* sp. | Ceraphronoidea | KF015227 | 16315 | 83.0 | 0.032 | -0.296 |
| *Ibalia leucospoides* | Cynipoidea | NC_026832 | 17212 | 86.3 | -0.032 | -0.012 |
| *Evania appendigaster* | Evanioidea | FJ593187 | 17817 | 77.8 | 0.027 | -0.349 |
| *Gasteruption* sp*.* | Evanioidea | KJ619460 | 17884 | 82.3 | 0.085 | -0.382 |
| *Pristaulacus compressus* | Evanioidea | KF500406 | 15563 | 84.0 | -0.087 | -0.306 |
| *Cotesia vestalis* | Ichneumonidea | NC_014272 | 15543 | 87.2 | -0.089 | 0.099 |
| *Diadegma semiclausum* | Ichneumonidea | EU871947 | 18728 | 87.4 | 0.009 | -0.198 |
| *Megalyra* sp*.* | Megalyroidea | KJ577600 | 18996 | 82.4 | -0.034 | 0.206 |
| *Spathius agrili* | Ichneumonidea | NC_014278 | 15425 | 84.0 | -0.074 | 0.182 |
| *Ceratobaeus* sp*.* | Platygastroidea | KF696669 | 15850 | 77.6 | 0.054 | -0.252 |
| *Idris* sp*.* | Platygastroidea | KF696670 | 15136 | 81.2 | 0.035 | -0.314 |
| *Pelecinus polyturator* | Proctotrupoidea | NC_026865 | 14896 | 81.2 | -0.031 | -0.341 |
| *Vanhornia eucnemidarum* | Proctotrupoidea | NC_008323 | 16567 | 80.1 | 0.086 | -0.328 |
| *Philanthus triangulum* | Sphecoidea | JN871914 | 16029 | 83.6 | 0.062 | -0.249 |
| *Orthogonalys pulchella* | Trigonaloidea | NC_025289 | 17277 | 83.8 | -0.066 | 0.210 |
| *Abispa ephippium* | Vespoidea | NC_011520 | 16953 | 80.6 | -0.019 | -0.380 |
| *Formica fusca* | Vespoidea | NC_026132 | 16673 | 83.4 | -0.033 | -0.322 |
| *Formica selysi* | Vespoidea | NC_026711 | 16752 | 83.3 | -0.031 | -0.324 |
| *Leptomyrmex pallens* | Vespoidea | NC_023093 | 15588 | 69.5 | 0.062 | -0.445 |
| *Myrmica scabrinodis* | Vespoidea | NC_026133 | 15310 | 82.2 | -0.038 | -0.266 |
| *Pristomyrmex punctatus* | Vespoidea | NC_015075 | 16180 | 79.7 | -0.021 | -0.401 |
| *Solenopsis geminata* | Vespoidea | NC_014669 | 15552 | 76.5 | 0.009 | -0.445 |
| *Solenopsis invicta* | Vespoidea | NC_014672 | 15549 | 77.2 | -0.002 | -0.447 |
| *Solenopsis richteri* | Vespoidea | NC_014677 | 15560 | 76.9 | -0.002 | -0.439 |
| *Vespa mandarinia* | Vespoidea | NC_027172 | 15902 | 79.4 | -0.021 | -0.410 |
| *Wallacidia oculata* | Vespoidea | FJ611801 | 18442 | 77.4 | 0.132 | -0.286 |
| *Allantus luctifer* | Tenthredinoidea | NC_024664 | 15418 | 81.1 | 0.036 | -0.201 |
| *Cephus cinctus* | Cephoidea | FJ478173 | 19339 | 82.0 | 0.034 | -0.286 |
| *Calameuta idolon* | Cephoidea | NC_028446.1 | 19,746 | 79.6 | 0.048 | -0.275 |
| *Calameuta filiformis* | Cephoidea | NC_028445.1 | 20,055 | 79.0 | 0.053 | -0.280 |
| *Cephus pygmeus* | Cephoidea | KM377623.1 | 16,145 | 79.8 | 0.015 | -0.284 |
| *Orussus occidentalis* | Orussoidea | NC_012689 | 15947 | 76.2 | 0.017 | -0.308 |
| *Megaphragma amalphitanum* | Chalcidoidea | NC_028196 | 15041 | 85.3 | 0.071 | -0.185 |
| *Trichogramma japonicum* | Chalcidoidea | KU577436 | 15962 | 84.9 | 0.131 | -0.152 |
| *Trichogramma ostriniae* | Chalcidoidea | KU577437 | 16472 | 85.4 | 0.101 | -0.151 |

**Table S2 Base composition of the mitochondrial genomes in *Trichogramma japonicum* and *T. ostriniae***

| Gene | *Trichogramma japonicum* | | | | | | *Trichogramma ostriniae* | | | | | | | | |
| --- | --- | --- | --- | --- | --- | --- | --- | --- | --- | --- | --- | --- | --- | --- | --- |
|  | T% | C% | A% | G% | (A+T)% | AT-skew | GC-skew |  | T% | C% | A% | G% | (A+T)% | AT-skew | GC-skew |
| Entire Genome | 48.50 | 7.40 | 34.70 | 9.40 | 83.20 | -0.1659 | -0.1190 |  | 49.00 | 7.50 | 34.00 | 9.50 | 83.00 | -0.1807 | 0.1176 |
| All protein-coding genes | 34.05 | 9.47 | 49.03 | 7.45 | 83.08 | 0.1803 | -0.1194 |  | 34.75 | 9.37 | 48.50 | 7.38 | 83.25 | 0.1652 | -0.1188 |
| *atp6* | 48.44 | 9.93 | 32.59 | 9.04 | 81.04 | -0.1956 | -0.0469 |  | 48.15 | 9.33 | 33.48 | 9.04 | 81.63 | -0.1797 | -0.0161 |
| *atp8* | 50.00 | 5.95 | 39.88 | 4.17 | 89.88 | -0.1126 | -0.1765 |  | 52.38 | 7.14 | 36.90 | 3.57 | 89.29 | -0.1733 | -0.3333 |
| *cob* | 41.75 | 11.49 | 36.05 | 10.70 | 77.81 | -0.0733 | -0.0356 |  | 42.72 | 11.32 | 35.96 | 10.00 | 78.68 | -0.0858 | -0.0617 |
| *cox1* | 45.70 | 10.48 | 30.34 | 13.48 | 76.04 | -0.2021 | 0.1250 |  | 45.44 | 10.22 | 30.92 | 13.41 | 76.37 | -0.1901 | 0.1350 |
| *cox2* | 45.37 | 7.78 | 35.10 | 11.75 | 80.47 | -0.1277 | 0.2030 |  | 45.23 | 7.49 | 36.56 | 10.72 | 81.79 | -0.1059 | 0.1774 |
| *cox3* | 48.99 | 8.96 | 29.80 | 12.25 | 78.79 | -0.2436 | 0.1548 |  | 47.98 | 8.46 | 32.32 | 11.24 | 80.30 | -0.1950 | 0.1410 |
| *nad1* | 49.25 | 6.62 | 33.33 | 10.79 | 82.59 | -0.1928 | 0.2393 |  | 47.44 | 7.26 | 34.40 | 10.90 | 81.84 | -0.1593 | 0.2000 |
| *nad2* | 51.90 | 4.00 | 38.90 | 5.20 | 90.80 | -0.1432 | -0.1304 |  | 52.60 | 4.40 | 38.70 | 4.30 | 91.30 | -0.1522 | -0.0115 |
| *nad3* | 50.41 | 5.51 | 34.71 | 9.37 | 85.12 | -0.1845 | 0.2593 |  | 49.44 | 5.56 | 37.50 | 7.50 | 86.94 | -0.1374 | 0.1489 |
| *nad4* | 53.27 | 5.80 | 31.85 | 9.08 | 85.12 | -0.2517 | 0.2200 |  | 51.49 | 6.32 | 32.96 | 9.23 | 84.45 | -0.2194 | 0.1866 |
| *nad4l* | 55.90 | 3.13 | 33.33 | 7.64 | 89.24 | -0.2529 | 0.4194 |  | 57.29 | 2.08 | 31.60 | 9.03 | 88.89 | -0.2891 | 0.6250 |
| *nad5* | 52.55 | 5.75 | 33.33 | 8.36 | 85.88 | -0.2238 | 0.1849 |  | 51.93 | 5.53 | 33.93 | 8.62 | 85.86 | -0.2097 | 0.2185 |
| *nad6* | 45.94 | 5.35 | 44.73 | 3.97 | 90.67 | -0.0133 | -0.1481 |  | 45.72 | 5.93 | 44.33 | 4.01 | 90.05 | -0.0155 | -0.1930 |
| *rrnL* | 46.00 | 4.57 | 42.36 | 7.07 | 88.36 | -0.0412 | 0.2147 |  | 44.26 | 4.83 | 43.75 | 7.17 | 88.00 | -0.0058 | 0.1951 |
| *rrnS* | 47.34 | 4.56 | 40.38 | 7.72 | 87.72 | -0.0794 | 0.2577 |  | 44.39 | 4.26 | 44.13 | 7.23 | 88.52 | -0.0029 | 0.2584 |
| A+T-rich region | 45.19 | 4.72 | 45.87 | 4.22 | 91.06 | 0.0074 | -0.0566 |  | 45.30 | 4.94 | 43.73 | 6.02 | 89.04 | -0.0176 | 0.0989 |

**Table S3 Codon usage of the *Trichogramma* *ostriniae* and *T.* *japonicum*** mitochondrial genome

| *Trichogramma ostriniae* | | | | | | | | | | | |  | *Trichogramma japonicum* | | | | | | | | | | | |
| --- | --- | --- | --- | --- | --- | --- | --- | --- | --- | --- | --- | --- | --- | --- | --- | --- | --- | --- | --- | --- | --- | --- | --- | --- |
| AA | Codon | No. | RSCU | AA | Codon | No. | RSCU | AA | Codon | No. | RSCU |  | AA | Codon | No. | RSCU | AA | Codon | No. | RSCU | AA | Codon | No. | RSCU |
| Phe | UUU | 423 | 1.96 | Ser | UCU | 120 | 2.9 | Tyr | UAU | 190 | 1.91 |  | Phe | UUU | 417 | 1.92 | Ser | UCU | 129 | 3.06 | Tyr | UAU | 195 | 1.93 |
|  | UUC | 8 | 0.04 |  | UCC | 2 | 0.05 |  | UAC | 9 | 0.09 |  |  | UUC | 18 | 0.08 |  | UCC | 2 | 0.05 |  | UAC | 7 | 0.07 |
| Leu | UUA | 537 | 5.56 |  | UCA | 88 | 2.13 | TER | UAA | 0 | 0 |  | Leu | UUA | 550 | 5.67 |  | UCA | 85 | 2.02 | TER | UAA | 0 | 0 |
|  | UUG | 23 | 0.24 |  | UCG | 5 | 0.12 |  | UAG | 0 | 0 |  |  | UUG | 14 | 0.14 |  | UCG | 0 | 0 |  | UAG | 0 | 0 |
|  | CUU | 14 | 0.14 | Pro | CCU | 60 | 2.4 | His | CAU | 56 | 1.93 |  |  | CUU | 12 | 0.12 | Pro | CCU | 68 | 2.75 | His | CAU | 50 | 1.69 |
|  | CUC | 0 | 0 |  | CCC | 1 | 0.04 |  | CAC | 2 | 0.07 |  |  | CUC | 1 | 0.01 |  | CCC | 2 | 0.08 |  | CAC | 9 | 0.31 |
|  | CUA | 6 | 0.06 |  | CCA | 38 | 1.52 | Gln | CAA | 38 | 1.77 |  |  | CUA | 4 | 0.04 |  | CCA | 29 | 1.17 | Gln | CAA | 31 | 1.55 |
|  | CUG | 0 | 0 |  | CCG | 1 | 0.04 |  | CAG | 5 | 0.23 |  |  | CUG | 1 | 0.01 |  | CCG | 0 | 0 |  | CAG | 9 | 0.45 |
| Cys | UGU | 31 | 2 | Ile | AUU | 437 | 1.99 | Thr | ACU | 61 | 2.16 |  | Cys | UGU | 28 | 2 | Ile | AUU | 421 | 1.98 | Thr | ACU | 74 | 2.49 |
|  | UGC | 0 | 0 |  | AUC | 2 | 0.01 |  | ACC | 4 | 0.14 |  |  | UGC | 0 | 0 |  | AUC | 5 | 0.02 |  | ACC | 2 | 0.07 |
| Trp | UGA | 61 | 1.72 | Met | AUA | 336 | 1.85 |  | ACA | 46 | 1.63 |  | Trp | UGA | 66 | 1.86 | Met | AUA | 332 | 1.85 |  | ACA | 42 | 1.41 |
|  | UGG | 10 | 0.28 |  | AUG | 28 | 0.15 |  | ACG | 2 | 0.07 |  |  | UGG | 5 | 0.14 |  | AUG | 27 | 0.15 |  | ACG | 1 | 0.03 |
| Arg | CGU | 18 | 1.57 | Val | GUU | 85 | 2.39 | Ala | GCU | 48 | 2.67 |  | Arg | CGU | 22 | 1.96 | Val | GUU | 103 | 2.61 | Ala | GCU | 53 | 2.9 |
|  | CGC | 1 | 0.09 |  | GUC | 1 | 0.03 |  | GCC | 3 | 0.17 |  |  | CGC | 0 | 0 |  | GUC | 1 | 0.03 |  | GCC | 2 | 0.11 |
|  | CGA | 26 | 2.26 |  | GUA | 50 | 1.41 |  | GCA | 21 | 1.17 |  |  | CGA | 21 | 1.87 |  | GUA | 49 | 1.24 |  | GCA | 18 | 0.99 |
|  | CGG | 1 | 0.09 |  | GUG | 6 | 0.17 |  | GCG | 0 | 0 |  |  | CGG | 2 | 0.18 |  | GUG | 5 | 0.13 |  | GCG | 0 | 0 |
| Asn | AAU | 258 | 1.9 | Ser | AGU | 36 | 0.87 | Gly | GGU | 64 | 1.61 |  | Asn | AAU | 247 | 1.89 | Ser | AGU | 44 | 1.04 | Gly | GGU | 79 | 1.99 |
|  | AAC | 13 | 0.1 |  | AGC | 1 | 0.02 |  | GGC | 4 | 0.1 |  |  | AAC | 14 | 0.11 |  | AGC | 1 | 0.02 |  | GGC | 2 | 0.05 |
| Lys | AAA | 122 | 1.79 |  | AGA | 76 | 1.84 |  | GGA | 73 | 1.84 |  | Lys | AAA | 124 | 1.85 |  | AGA | 66 | 1.57 |  | GGA | 63 | 1.58 |
|  | AAG | 14 | 0.21 |  | AGG | 3 | 0.07 |  | GGG | 18 | 0.45 |  |  | AAG | 10 | 0.15 |  | AGG | 10 | 0.24 |  | GGG | 15 | 0.38 |
| Asp | GAU | 50 | 1.72 | Glu | GAA | 68 | 1.86 |  |  |  |  |  | Asp | GAU | 61 | 1.97 | Glu | GAA | 58 | 1.61 |  |  |  |  |
|  | GAC | 8 | 0.28 |  | GAG | 5 | 0.14 |  |  |  |  |  |  | GAC | 1 | 0.03 |  | GAG | 14 | 0.39 |  |  |  |  |

RSCU: Relative synonymous codon usage; AA: Amino acid; No.: Number.

**Table S4 Primers used in this study for amplify the mitochondrial genome sequences of *Trichogramma* *japonicum* and *T.* *ostriniae***

| Species | Amplified region | Name | The sequence of primers |
| --- | --- | --- | --- |
| Both species | Partial *cox1* | 1718-COI-F | 5’-GGAGGATTTGGAAATTGATTAGTTCC-3’ |
|  |  | 2191-COI-R | 5’-CCCGGTAAAATTAAAATATAAACTTC-3’ |
| *T.* *japonicum* | Complete genome except for partial *cox1* | Tj-COI-F | 5' -TCTTTACATATTGCTGGGGTT- 3' |
|  |  | Tj-COI-R | 5'- TATTTATTCGAGGAAAAGCCAT- 3' |
| *T. ostriniae* | Complete genome except for partial *cox1* | To-COI-F | 5' -TCAAATTTATCTCATAGGGGTC- 3' |
|  |  | To-COI-R | 5' -TTCGAGGAAAAGCTATATCAGG- 3' |


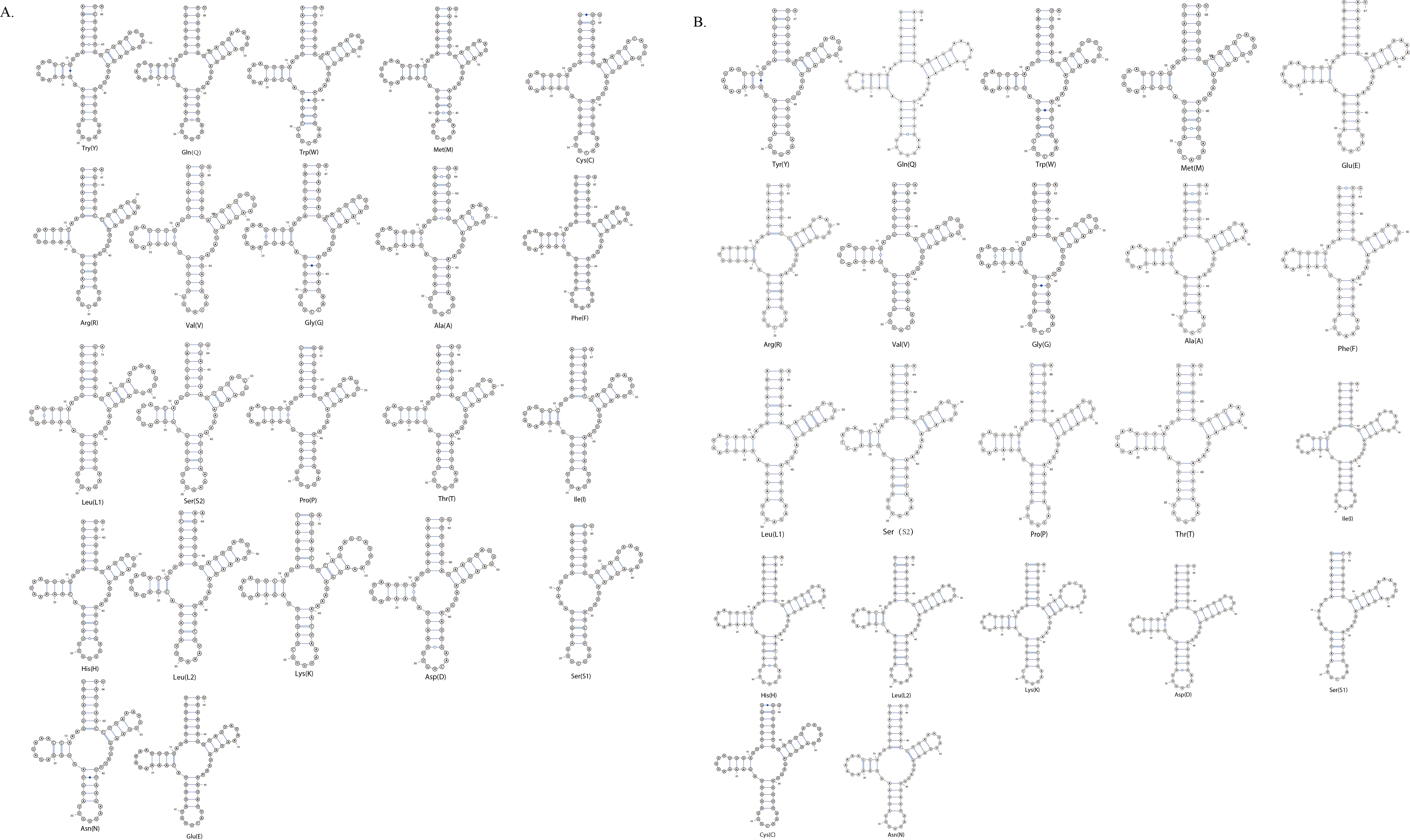


**Figure S1 Predicted secondary clover-leaf structures for the 22 tRNA genes in *Trichogramma ostriniae* (A) and *T. japonicum* (B)**


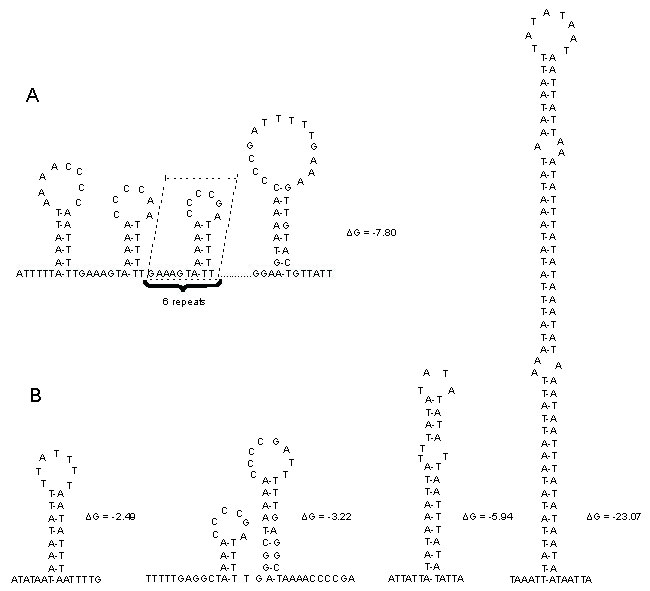


**Figure S2 Identified stem and loop structures in the control region of *Trichogramma ostriniae* (A) and *T. japonicum* (B)**


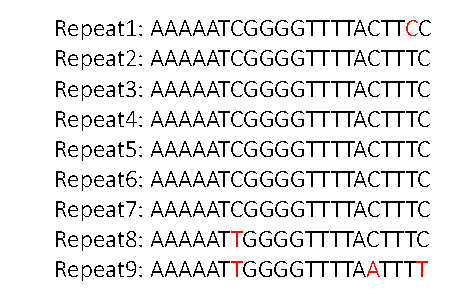


**Figure S3 Alignment of nine repeat elements in the A+T-rich region of *Trichogramma ostriniae* mitochondrial genome**

**Appendix S1** Input file for MLGO analysis and phylogenetic relationships within Chalcoidea using gene order

**Input data (genes from 1-37 are as in Ancestral pattern of insect mitochondrial gene arrangement of Figure 1):**

>ChalcidoidGO

31 32 -33 -34 -35 -36 -37 38 3 1 -2 5 -7 -6 -17 -16 -15 -14 -13 -12 -11 -10 -9 -8 18 19 20 21 22 -23 -24 -25 -26 -27 28 -29 30 $

>Trichogramma

31 32 -33 -34 -35 -18 -16 -37 -36 -19 -17 -3 -5 -4 -2 -7 -8 22 -23 -1 -21 -20 -6 -15 -14 -13 -12 -11 -10 -9 -24 -25 -26 -27 28 -29 30 $

>Megaphragma

31 32 -33 -34 -35 -18 -19 -2 -17 -37 -36 -16 -3 20 21 7 -5 -4 -1 -6 -15 -14 -13 -12 -11 -10 -9 -8 22 -23 -24 -25 -26 -27 28 -29 30 $

>Philotrypesis

31 32 -1 -4 5 -7 -6 -17 -2 -33 -34 -18 -37 -15 -14 -13 -12 -11 -10 -9 -8 22 -23 -24 -25 -26 -27 28 -29 30 $

>Nasonia

31 -4 5 -7 20 -33 -34 -35 -18 -37 -17 -16 -15 -14 -13 -12 -11 -10 -9 -8 -23 -24 -25 -26 -27 28 -29 30 $

>A1

37 18 35 34 33 -20 7 2 17 6 -5 4 1 32 -31 30 29 -28 27 26 25 24 23 -22 8 9 10 -11 12 13 14 15 16 $

>A2

36 37 18 35 34 33 32 -31 30 29 -28 27 26 25 24 23 -22 8 9 10 -11 12 13 14 15 6 1 4 5 -7 -21 -20 -19 -2 3 16 17 $

>A3

36 37 17 2 19 18 35 34 33 32 -31 30 29 -28 27 26 25 24 23 -22 8 9 10 -11 12 13 14 15 6 1 4 5 -7 -21 -20 3 16 $

**Inferred tree:**


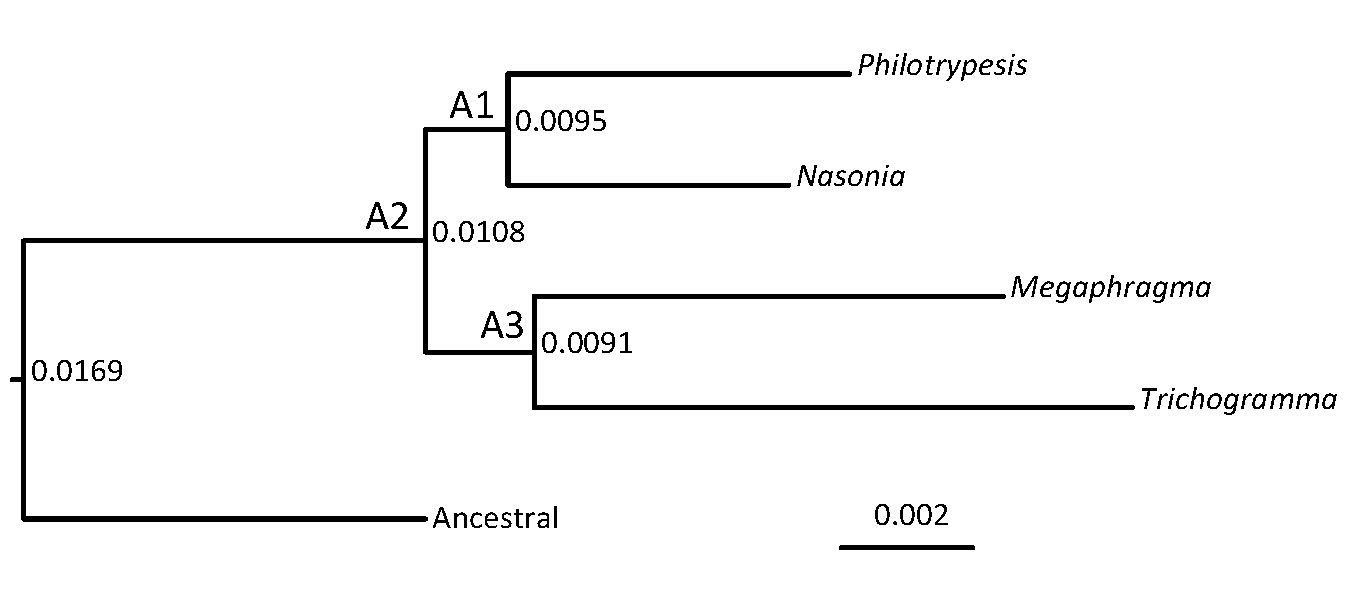

Supplement: Supplementary file 1 — Supplemental information [file 41598_2018_25338_MOESM1_ESM.doc]
